# Docosahexaenoic Acid Status in Pregnancy Determines the Maternal Docosahexaenoic Acid Status 3-, 6- and 12 Months Postpartum. Results from a Longitudinal Observational Study

**DOI:** 10.1371/journal.pone.0136409

**Published:** 2015-09-02

**Authors:** Maria Wik Markhus, Josef Daniel Rasinger, Marian Kjellevold Malde, Livar Frøyland, Siv Skotheim, Hanne Cecilie Braarud, Kjell Morten Stormark, Ingvild Eide Graff

**Affiliations:** 1 National Institute of Nutrition and Seafood Research (NIFES), Bergen, Norway; 2 The Department of Biomedicine, Faculty of Medicine and Dentistry, University of Bergen, Bergen, Norway; 3 Regional Centre for Child and Youth Mental Health and Child Welfare, Uni Research Health, Bergen, Norway; Steno Diabetes Center, DENMARK

## Abstract

**Background:**

Essential fatty acid status as well as docosahexaenoic acid (DHA, 22:6n-3) declines during pregnancy and lactation. As a result, the DHA status may not be optimal for child development and may increase the risk for maternal postpartum depression. The objective of this study was to assess changes in the maternal fatty acid status from pregnancy to 12 months postpartum, and to study the impact of seafood consumption on the individual fatty acid status.

**Methods:**

Blood samples and seafood consumption habits (gestation week 28, and three-, six- and 12 months postpartum) were collected in a longitudinal observational study of pregnant and postpartum women (n = 118). Multilevel linear modeling was used to assess both changes over time in the fatty acid status of red blood cells (RBC), and in the seafood consumption.

**Results:**

Six fatty acids varied the most (>80%) across the four time points analyzed, including the derivative of the essential α-linoleic acid (ALA, 18:3n-3), DHA; the essential linoleic acid (LA, 18:2 n-6); and the LA derivative, arachidonic acid (AA, 20:4n-6). Over all, a large variation in individuals’ DHA- and AA status was observed; however, over the 15-month study period only small inter-individual differences in the longitudinal trajectory of DHA- and AA abundance in the RBC were detected. The median intake of seafood was lower than recommended. Regardless, the total weekly frequency of seafood and eicosapentaenoic acid (EPA, 20:5n-3)/DHA-supplement intake predicted the maternal level of DHA (μg/g RBC).

**Conclusion:**

The period of depletion of the maternal DHA status during pregnancy and lactation, seem to turn to repletion from about six months postpartum towards one year after childbirth, irrespective of RBC concentration of DHA during pregnancy. Seafood and EPA/DHA-supplement intake predicted the DHA levels over time.

**Trial Registration:**

www.helseforskning.etikkom.no 2009/570/REC, project number: 083.09

## Introduction

Pregnancy and lactation are periods of increased nutritional vulnerability as nutrient needs are increased. To maintain the delicate balance between the needs of the mother and those of the fetus an adequate supply of nutrients is required. Some of the nutrients required protect maternal health while others affect birth outcome and infant health.

Inadequate nutrient supply may in the worst case cause a biological competition between the mother and the fetus [1], and maintaining or regaining a good nutrition status during pregnancy is therefore important. This is also true after birth, as adequate maternal nutrient stores is necessary for both lactation and general good health, as well as preconceptionally for a possible new pregnancy.

The n-3 fatty acid docosahexaenoic acid (DHA, 22:6n-3), and the n-6 fatty acid, arachidonic acid (AA, 20:4n-6), derived from the essential fatty acids α-linoleic acid (ALA, 18:3n-3) and linoleic acid (LA, 18:2n-6) respectively, are fundamental structural components in the brain and the central nervous system and play an important role in the growth, development and structure of the brain [2]. Pregnancy is associated with a decrease in the DHA and AA status, and normalization postpartum is slow [3, 4]. A low seafood intake in general has been associated with both higher rates of major depression [5] and postpartum depression [6], and recently a low n-3 index [7] in pregnancy was suggested as a possible biological risk factor for postpartum depression [8], which subsequently can affect mother-infant interaction in a negative way [9].

During the last trimester of fetal life and the first two years of childhood, the brain undergoes a period of rapid growth termed the “brain growth spurt”. Lower fish consumption in pregnancy has been associated with suboptimum neurodevelopmental outcomes in children [10] and higher fish consumption in pregnancy has been associated with better infant cognition [11]. Further, a higher maternal DHA status in pregnancy has been associated with better infant problem solving at 12 months (unpublished observations, Braarud, H. *et al*, 2014). In addition to dietary intakes, *FADS* genotype influence DHA amounts in maternal RBC and might affect the child’s DHA supply during pregnancy [12].

Seafood is a unique dietary source of important nutrients such as eicosapentaenoic acid (20:5n-3) EPA and DHA, vitamin D and B_12_, and the trace minerals iodine and selenium [13]. Both lean and oily fish is improving the overall nutrient content of the diet and are consequently regarded as a natural part of a healthy, balanced diet [14]. Inadequate supply of essential micronutrients in this period may compromise brain function. The general recommendation is to eat 2–3 portions of fish a week [15, 16]. Women are recommended to continue their fish consumption during pregnancy [11, 17] and to consume a minimum of 200 mg DHA daily [18]. However, national surveys in Norway show an increasing intake of red meat and a constant low intake of seafood over the past decades [14, 19–21].

The aim of the present study was to conduct a longitudinal analysis to investigate changes in the total fatty acid status during pregnancy and the first year postpartum, within a population of Norwegian mothers. We also aim to elucidate the impact seafood consumption has on the fatty acid status of red blood cells (RBC) in the same study population.

## Subjects and Methods

### Study population and design

The investigation originated from a community based study with a prospective cohort design performed in a municipality outside Bergen, Norway. The main objectives of the cohort were to study the associations between seafood consumption, mental health, and infant development. Enrolment was open for 20 months and the source population was all women, pregnant in their 24^th^ week of gestation, between November 2009 and June 2011, in the municipality. Midwifes or medical doctors recruited the women at a routine visit in the 24^th^ week of gestation. At the routine visit 2 and 6 weeks postpartum public health nurses recruited women who had already given birth when the enrolment started. Thus, participation after time point 1 is larger. The original cohort comprised four waves of follow-up: in the 28^th^ gestational week (T1), and at three- (T2), six- (T3), and 12 (T4) months postpartum. Exclusion criteria were lack of Norwegian language skills or/and premature childbirth (over four weeks). Blood samples (non-fasting venous) were drawn from the participants at all time points. The study design has challenges due to recruitment at both T1 and T2. The reason for this was due to practical issues in the recruitments process in the municipality where the source population originated. The initial plan was that family doctors was going to recruit pregnant women to the study at the routine visit in gestational week 24. However, over 50% of the pregnant women had their routine visits with their midwives at the well-baby clinic in gestational week 24. To ensure that all women in the source population was asked to participate and minimize selection bias, public health care nurses started recruiting women that had already just given birth.

**Fig 1:**
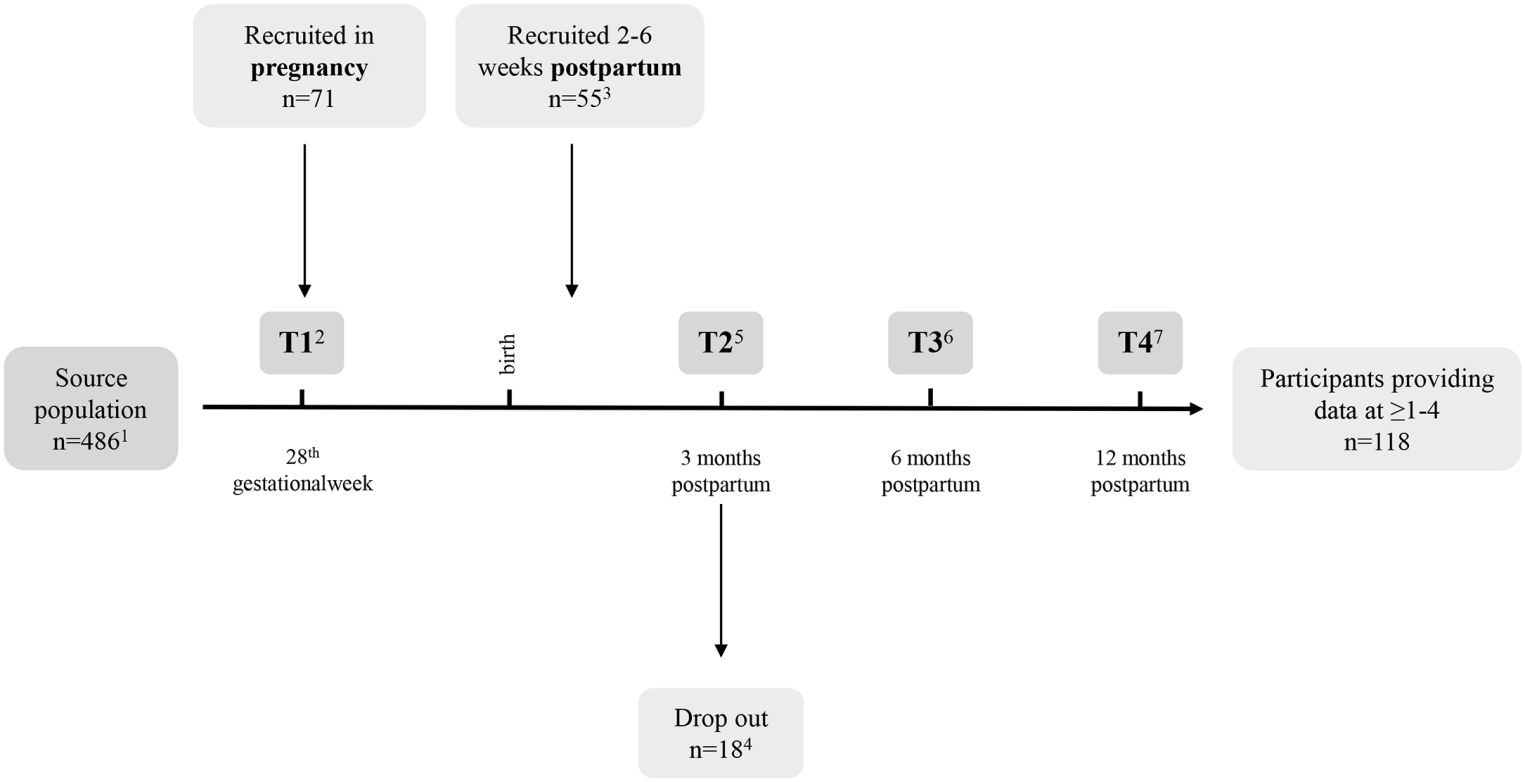
Flow chart of study- population and design. ^1^ Not all women from the source population were asked to participate in the study while still pregnant, partly for practical reasons and partly due to exclusion criteria (unable to understand Norwegian). ^2^ 2 BS and 16 FFQ missing at random or passive dropout. ^3^ Women recruited 2–6 weeks postpartum to restrict selection bias as not the whole source population was asked to participate during pregnancy due to practical reasons. ^4^ Passive drop-out sometime between T1 and T2. ^5^ 18 BS and 16 FFQ missing at random or passive drop-out. ^6^ 21 BS and 21 FFQ missing at random or passive drop-out. ^7^ 30 BS and 26 FFQ missing at random or passive drop-out. BS: blood sample; FFQ: food frequency questionnaire.

Fig 1 represents a flow chart of the study design, participation and data collected at each time point.

All volunteers provided written informed consent. Participants could withdraw from the study at any time, without reason. The procedures followed were in accordance with the Helsinki Declaration of 1975 (revised in 2008), and approved by the Regional Committee of Ethics in Medical Research West and the Norwegian Social Science Data Services. A research specific biobank was established and approved for storage of biological samples.

### Blood sampling and biomarker analysis

For preparation of red blood cells (RBC) venous blood from the elbow cavity was collected in ice water cooled 4 ml BD Vacutainer K2E 7.2 mg vials. Blood collected in K2E vials was centrifuged (10 min, 1000 g, RT) within 30 minutes. RBCs were adequately separated to ensure a clean blood fraction. Blood samples were stored at -20°C for 0–4 weeks prior to transportation on dry ice to a -80°C freezer where they were stored until analysis.

The fatty acid (FA) composition of total RBC was determined by ultrafast gas chromatography (UFGC) (Thermo Electron Corporation, Massachusetts, USA), a method developed by Araujo *et al*. [22]. After direct methylation of FA in homogenized samples, boron trifluoride (BF_3_) and internal standard (19:0 methyl ester) were added, followed by extraction with hexane. The FA composition was calculated using an integrator (Chromeleon 6.80, Dionex Corporation, California, USA), connected to the UFGC and identification ascertained by standard mixtures of methyl esters (Nu-Chek, Minnesota, USA). Limit of quantification was 0.01 mg FA/g samples (wet weight). The analytical quality of the method and systematic errors were controlled by the certified reference materials (CRM) CRM 162 (soy oil) and CRM 163 (pig fat). Results are expressed as relative (% w/w) and absolute amounts (μg/g).

### Seafood food frequency questionnaire and other variables

A questionnaire designed for the study was sent by e-mail to all participants in the 28^th^ gestational week (T1) and three moths postpartum (T2), and by postal mail six- (T3) and twelve months postpartum (T4). Questions regarding the seafood consumption were assessed using a semi-quantitative seafood food frequency questionnaire (FFQ). The FFQ was designed to capture the habitual intake of seafood and the use of dietary supplements [23]. To enable aggregation and quantity estimation of the individual seafood consumption, ordinal data from the seafood-FFQ was converted to numerical data, using the seafood-index system [24]. The Seafood index was established based on the mean weekly intake of seafood eaten as dinner and as spread (never = 0, <1 time/month = 0.15, 1–3 times/month = 0.5, 1–2 times/week = 1.5, ≥3 times/week = 3). Omega-3 supplement intake was re-coded from general frequency to frequency of average weekly intake in order to correlate and estimate intake in conjunction with the seafood intake. A numerical index was assigned, similar to that for seafood (never = 0, 1–3 times/month = 0.5, 1–3 times/week = 1, 4–7 times/week = 5). An index termed the total seafood index was computed as a sum of the participants’ seafood dinner index, seafood spread index and supplement index in order to capture a crude total intake of EPA and DHA from seafood and omega-3 supplements. Further details regarding the FFQ and the seafood-index have been explained in detail elsewhere [24]. In the same questionnaire, participants provided data on age, income, anthropometric measures, education, marital status and lifestyle factors.

Breastfeeding habits were assessed through a combination of a 24-hour dietary recall interview and an interviewer administered FFQ. The infant FFQ included questions on the frequency of breast milk feeding, followed by questions on exclusive breastfeeding. Breastfeeding was categorized in terms of yes/no based on daily breast feedings.

### Statistical analyses

Descriptive statistics and analysis of differences between participants recruited at two different time points were calculated using the Statistical Package for the Social Sciences (IBM SPSS Statistics 21, IBM Corporation, Norway). Data was tested for normality using the Shapiro-Wilk test. Potential differences in fatty acid status, total seafood and fish oil supplementation, descriptive, socioeconomic- and behavioral characteristics were assessed using dependent t-tests if the assumption of normality was met; otherwise the Man-Whitney test was employed. Relative and absolute amounts of the FA as measured in RBC are presented as means (± 95% confidence intervals, CI). Independent sample t-tests were performed to assess if mean values of FA of participants whom we sampled blood from at all four time points were significantly different to those we did not sample blood from at all four timepointsat each time point.

Qlucore Omics Explorer version 2.3 (Qlucore AB, Lund, Sweden) was used to perform unsupervised principal component analysis (PCA). For PCA analysis, each variable was standardized by subtraction of its mean value and division by its standard deviation across all samples. Variance filtering was applied to limit the number of FA in the standardized data set to the ones explaining >80% of the variation in the data. Heat maps were generated to visualize the abundance values of the FA, detected > limit of quantification for FA determination by UFGC, over time. The time course of the FA was analyzed further using multilevel linear models in the statistical package R (version 3.0.2) emulating the modelling approaches described in [25–27]. In brief, four statistical models were created: (i) a basic fixed intercept model predicting the FA status from the intercept only; (ii) a random intercept model predicting the FA status from the intercept allowing the intercepts to vary across people; (iii) a level 1 unconditional linear growth model with a random intercept and time as predictor and; (iv) a level 2 unconditional linear growth model with a random intercept and slope and time as predictor. In a final step (v), different time trajectories (second and third order polynomial) were added to the models. The models produced were compared to each other using Log likelihood tests and best fitting and most parsimonious model was chosen for further analyses in which the following predictors were added: (i) Seafood consumption as estimated from the Seafood FFQ to continuous variables, the total seafood-index [8]; (ii) the participants’ number of children which included the child born during the study; (iii) age; and (iv) education level, which were taken from the electronic questionnaire at T2. Data from T1 or T4 were used if records were missing. The FA raw data is presented in spaghetti plots on to which a locally weighted regression (lowess) line and population mean at each time point were superimposed. Relative to traditional techniques for the analysis of longitudinal data (e.g., ANOVA), multilevel linear modelling, offers many advantages including analysis at the individual level, complete flexibility in the responses to missing data, ability to handle clustered data, and generalization to non-normally distributed outcomes [26].

## Results

### Characteristics of study population

Descriptive characteristics of the study population at all four time points are presented as cross section data in Table 1. The participants’ number of children, including the child born in this study, was 1.8±1.2, range 1–7. The overall education level was high, and the income was average for Norway.

**Table 1:** Descriptive, socio-economic and behavioral characteristics of study participants (T1 n = 55, T2 n = 92, T3 = 82 and T4 = 78).

Characteristics	T1 Count (%)^1^	T2 Count (%)^1^	T3 Count (%)^1^	T4 Count (%)^1^	Difference between recruitment time point*
Age (mean ± SD)	30±4.8	31±5.3	31±5.5	32±5.5	p < 0.05
BMI (mean ± SD)	24±3.9^2^	25±4.8	25±4.4	24±4.2	*ns*
Education					*ns*
Junior high school	0 (0)	4 (4.3)	5 (6.1)	6 (7.7)	
High school	16 (29.1)	29 (31.5)	27 (32.9)	25 (32.1)	
< 4 years of university education^3^	24 (43.6)	34 (37.0)	35 (42.7)	35 (44.9)	
≥ 4 years of university education^3^	15 (27.3)	25 (27.2)	15 (18.3)	12 (15.4)	
Employment					*ns*
Full-time (80–100%)	48 (87.3)	78 (84.8)			
Part-time (50–79%)	2 (3.6)	6 (6.5)	NA^4^	NA^4^	
Part-time (<50%)	1 (1.8)	0 (0)			
Homemaker	1 (1.8)	2 (2.2)			
Other	3 (5.5)	6 (6.5)			
Marital status					*ns*
Married	23 (43.4)^1^	43 (46.7)	NA^4^	NA^4^	
Cohabiting	28 (52.8)	46 (50.0)			
Single	2 (3.8)	3 (3.3)			
Own income in NOK^5^					*ns*
No income	0 (0)	2 (2.2)			
< 150.000	2 (3.6)	6 (6.5)			
150.000–199.999	1 (1.8)	3 (3.3)	NA^4^	NA^4^	
200.000–299.999	12 (21.8)	11 (12.0)			
300.000–399.999	27 (49.1)	47 (51.1)			
400.000–499.999	10 (18.2)	14 (15.2)			
> 500.000 NOK	3 (5.5)	9 (9.8)			
Breastfeeding					*ns*
Yes	NA	79 (81.4)	66 (70.2)	27 (32.5)	
No		18 (18.6)	28 (29.8)	56 (67.5)	

* An independent sample t-test was applied to normal data (age) and Man-Whitney for non-normal data (BMI, education, employment, marital status, own income and breastfeeding)

^1^ Age and BMI are presented as mean ± SD

^2^ BMI pre-pregnancy

^3^ University or University College

^4^ Data not requested at time point 3 and 4 (no new recruitment)

^5^ 100 000 NOK ≈ 14 000 EUR

### Fatty acid profile and heat map analysis

Means and 95% CI of all quantified FA at all four time points are presented in Table 2. Means and 95% CI of essential FA (LA, AA and DHA) from participants sampled blood from at all four time points and those not present at each time point are shown in Table 3. There were no significant differences in the mean values of those participants whom we sampled blood from at all four time points and those we did not at each time point.

**Table 2:** Fatty acid status of red blood cells in relative and absolute (μg/g) amounts measured in gestational week 28 (T1), 3- (T2), 6- (T3) and 12 months (T4) postpartum^1^. Different letter indicate statistically different values.

Fatty acids in red blood cells	T1 (n = 69) expressed as:		T2 (n = 90) expressed as:		T3 (n = 88) expressed as:		T4 (n = 78) expressed as:	
	% of total FA	μg / g RBC	% of total FA	μg / g RBC	% of total FA	μg / g RBC	% of total FA	μg / g RBC
	Mean ± 95% CI							
14:0	0.6 ± 0.0	19 ± 2^a^	0.4 ± 0.0	9 ± 1^b^	0.4 ± 0.0	8 ± 1 ^b^	0.4 ± 0.0	9 ± 1 ^b^
16:0 PA	23.0 ± 0.4	662 ± 26 ^a^	21.0 ± 0.3	515 ± 12 ^b^	21.0 ± 0.4	471 ± 13 ^c^	20.0 ± 0.6	469 ± 16 ^c^
17:0	0.4 ± 0.0	10 ± 1 ^a^	0.3 ± 0.0	7 ± 0 ^b,^ ^2^	0.3 ± 0.0	7 ± 0 ^b,^ ^2^	0.3 ± 0.0	7 ± 0 ^b,^ ^2^
18:0 SA	14.0 ± 0.4	390 ± 7 ^a^	16.0 ± 0.3	384 ± 10 ^a^	16.0 ± 0.4	350 ± 5 ^b^	15.0 ± 0.5	342 ± 11 ^b^
20:0	0.3 ± 0.0	9 ± 0 ^a^	0.4 ± 0.0	9 ± 1 ^a, b^	0.4 ± 0.0	8 ± 0 ^b^	0.4 ± 0.0	9 ± 0 ^a^
22:0	0.7 ± 0.0	19 ± 1	1.0 ± 0.1	25 ± 2	1.0 ± 0.0	24 ± 2	1.1 ± 0.0	25 ± 1
24:0	0.1 ± 0.1	2 ± 3 ^3^	0.3 ± 0.2	7 ± 4 ^2^	0.4 ± 0.1	7 ± 2 ^2^	0.0±0.0	0±0 ^3^
16:1^4^	0.9 ± 0.1	28 ± 4 ^a^	0.6 ± 0.0	14 ± 1 ^b^	0.5 ± 0.0	12 ± 2 ^b^	0.6 ± 0.0	15 ± 1 ^b^
18:1^4^	16.0 ± 0.4	452 ± 25 ^a^	14.0 ± 0.3	344 ± 10 ^b^	14.0 ± 0.4	320 ± 10 ^c^	13.0 ± 0.4	311 ± 12 ^c^
24:1n-9	1.7 ± 0.1	47 ± 2 ^a^	2.7 ± 0.1	65 ± 3 ^b^	1.9 ± 0.2	45 ± 5 ^a^	2.7 ± 0.1	62 ± 3 ^b^
18:2n-6 LA	13.0 ± 0.7	390 ± 33 ^a^	13.0 ± 0.4	303 ± 11 ^b^	12.0 ± 0.4	267 ± 10 ^c^	11.0 ± 0.5	254 ± 12 ^c^
16:3n-3	0 ± 0	0 ± 0 ^3^	0.4 ± 0.1	9 ± 1 ^2^	0.6 ± 0.1	15 ± 2	0.7 ± 0.1	16 ± 2
20:2n-6	0.3 ± 0.0	8 ± 1 ^a,^ ^2^	0.2 ± 0	5 ± 1 ^b,^ ^2^	0.2 ± 0.0	4 ± 1 ^b,^ ^2^	0.2 ± 0.0	4 ± 1 ^b,^ ^2^
20:3n-6	1.7 ± 0.1	48 ± 3 ^a^	0 ± 0	36 ± 2 ^b^	1.4 ± 0.1	31 ± 2 ^c^	0.2 ± 0.0	33 ± 2 ^c^
20:4n-6 AA	11.0 ± 0.4	320 ± 12 ^a^	13.0 ± 0.4	324 ± 11 ^a^	12.7 ± 0.4	286 ± 6 ^b^	12.0 ± 0.5	290 ± 12 ^b^
22:4n-6	1.9 ± 0.1	55 ± 3 ^a^	2.0 ± 0.1	50 ± 3 ^a, b^	2.0± 0.1	45 ± 3 ^b^	2.5 ± 0.3	57 ± 7 ^a^
21:5n-3	1.0 ± 0.2	38 ± 6 ^a^	1.0 ± 0.2	14 ± 5 ^b^	0.0 ± 0.0	0 ± 0 ^c,^ ^3^	0.1 ± 0.1	1 ± 1 ^c,^ ^3^
22:5n-6	0.3 ± 0.0	10 ± 1 ^a^	0.2 ± 0.0	6 ± 1 ^b,^ ^2^	1.0 ± 0.2	22 ± 5 ^c^	0.5 ± 0.1	11 ± 3 ^a^
18:3n-3 ALA	0.3 ± 0.0	9 ± 1 ^a, 2^	0 ± 0	1 ± 0 ^b,^ ^3^	0.1 ± 0.0	9 ± 11 ^a, b, c,^ ^2^	0.2 ± 0.0	4 ± 1 ^c,^ ^2^
20:5n-3 EPA	0.8 ± 0.1	22 ± 3 ^a, b^	1.0 ± 0.1	25 ± 3 ^a^	0.9 ± 0.1	20 ± 3 ^a, b^	0.8 ± 0.1	18 ± 2 ^a, b^
22:5n-3 DPA	1.8 ± 0.1	50 ± 3 ^a, b^	2.3 ± 0.1	55 ± 2 ^a^	2.0 ± 0.1	46 ± 2 ^b^	2.0 ± 0.1	46 ± 2 ^b^
22:6n-3 DHA	6.0 ± 0.3	164 ± 9 ^a^	6.0 ± 0.3	132 ± 7 ^b^	5.9 ± 0.3	137 ± 10 ^b^	7.2 ± 0.2	165 ± 7 ^a^

^1^ The fatty acids 6:0, 8:0, 10:0, 12:0, 16:2n-4, 20:3n-3, 16:4n3, 18:4n3 and 20:3n9 were quantified as 0**±**0 μg / g RBC at all time points. The fatty acids 20:4n-4 and 24:5n-3 were quantified as < 0**±**1 μg / g RBC at all time points. The fatty acid 18:3n-6 and 22:1 were quantified as < 1±3 0 μg / g RBC at all time points. The fatty 15:0 and the fatty 20:1 was quantified as < 5±2 μg / g RBC and < 7±5 μg / g RBC, respectively at all time points. AA: arachidonic acid; LA: linoleic acid; PA: palmitic acid; SA: stearic acid

^2^ Mean < the limit of quantification for the method (LOQ 10 μg / g RBC).

^3^ Mean < the limit of detection for the method (LOD 3 μg / g RBC).

^4^ The method does not specify monounsaturated fatty acids.

**Table 3:** Fatty acid status of red blood cells in relative and absolute (μg/g) amounts measured in gestational week 28 (T1), 3- (T2), 6- (T3) and 12 months (T4) postpartum, for linoleic acid (18:2n-6 LA) arachidonic acid (20:4n-6 AA) and docosahexaenoic acid (22:6n-3 DHA), comparing those participants that provided a sample at all four time points (A, n = 35) and those that did not (B).

	Fatty acids		n	Mean ± 95% CI	*p (2-tailed)* ^1^		Fatty acids		n	Mean ± 95% CI	*p (2-tailed)* ^1^
**T1 (n = 69)**	18:2n-6 LA %	A	35	13±8	*0*.*96*	**T2 (n = 90)**	18:2n-6 LA %	A	35	12±7	*0*.*51*
		B	34	13±11				B	55	13±5	
	18:2n-6 LA μg / g	A	35	396±42	*0*.*69*		18:2n-6 LA μg / g	A	35	301±18	*0*.*74*
		B	34	383±52				B	55	305±15	
	20:4n-6 AA %	A	35	11±6	*0*.*68*		20:4n-6 AA %	A	35	14±6	*0*.*09*
		B	34	11±5				B	55	13±5	
	20:4n-6 AA μg / g	A	35	323±17	*0*.*61*		20:4n-6 AA μg / g	A	35	336±18	*0*.*07*
		B	34	317±18				B	55	316±13	
	22:6n-3 DHA %	A	35	6±4	*0*.*95*		22:6n-3 DHA %	A	35	5±4	*0*.*99*
		B	34	6±5				B	55	5±4	
	22:6n-3 DHA μg / g	A	35	168±13	*0*.*42*		22:6n-3 DHA μg / g	A	35	133±10	*0*.*86*
		B	34	161±13				B	55	132±9	
**T3 (n = 88)**	18:2n-6 LA %	A	35	12±6	*0*.*99*	**T4 (n = 78)**	18:2n-6 LA %	A	35	11±5	*0*.*82*
		B	52	12±6				B	43	11±4	
	18:2n-6 LA μg / g	A	35	273±20	*0*.*39*		18:2n-6 LA μg / g	A	35	256±16	*0*.*78*
		B	52	264±11				B	43	259±13	
	20:4n-6 AA %	A	35	13±6	*0*.*80*		20:4n-6 AA %	A	35	13±4	*0*.*25*
		B	52	13±5				B	43	13±5	
	20:4n-6 AA μg / g	A	35	290±12	*0*.*29*		20:4n-6 AA μg / g	A	35	298±14	*0*.*44*
		B	52	283±8				B	43	290±15	
	22:6n-3 DHA %	A	35	6±6	*0*.*45*		22:6n-3 DHA %	A	35	7±3	*0*.*65*
		B	52	6±5				B	43	7±3	
	22:6n-3 DHA μg / g	A	35	140±16	*0*.*62*		22:6n-3 DHA μg / g	A	35	168±8	*0*.*85*
		B	52	135±14				B	43	167±9	

^1^ Independent sample t-test, significant level 0.05.

Six months after birth 33% of the participants were below the 2.5 percentile (113 μg/g RBC) for DHA (μg/g RBC), using T4 as reference. The corresponding fractions were 27% 3 months after birth and 7% in pregnancy.

**Fig 2:**
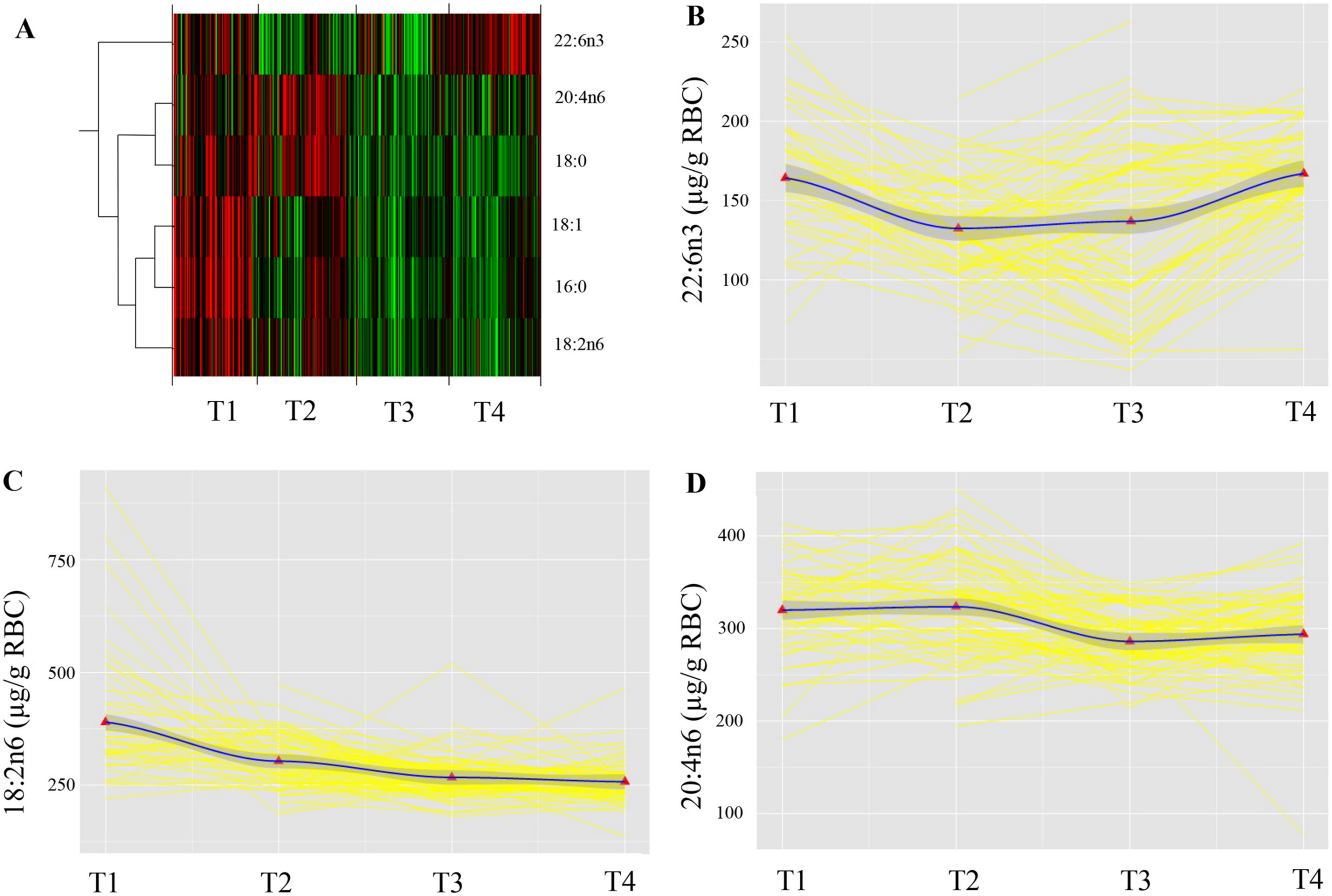
Repeated measurements of RBC FA status from pregnancy until 12 months postpartum. Panel A shows a heat map visualizing the abundance values of the FA across the repeated measurement. Red represents higher values and green represent lower values. A dendrogram on the left hand side of the heat map indicates similarity in the change over time in abundance profiles. Panel B, C and D represent raw data (yellow) of individually growth trajectories for **B**: 22:6n3 in RBC (μg/g); **C**: 18:2n6 RBC (μg/g); and **D**: 20:4n6 RBC (μg/g), measured in the 28^th^ gestational week (T1) and 3- (T2), 6- (T3) and 12 months (T4) postpartum. The black lines are locally weighted regression (lowess) lines that “smooth” the overall variability in each of the three datasets and provide an unbiased glimpse of the average trend in the data. The red triangles represent the population mean at each timepoint. T1 (28^th^ gestational week), T2 (three months postpartum), T3 (six months postpartum), T4 (twelve months postpartum).

The FA whose abundance profiles over time explained over 80% of the variation in the data were: palmitic acid (PA, 16:0), stearic acid (SA, 18:0), the sum of the monounsaturated 18:1 fatty acids, LA, AA and DHA (Fig 2A). Only essential FA and essential FA derivatives (AA and DHA) were subjected to further analyses using multilevel linear models.

### Multilevel linear modeling of DHA, LA and AA status

In this study, multilevel linear models or polynomials (linear, quadratic, cubic) were used to study the rate of change of fatty acid status in red blood cells over time, from pregnancy until 12 months postpartum. In addition, it was assessed if a statistical model can be found that best describes the change over time.

There were significant between-subject variations in average DHA status (μg/g RBC) over the time (data not shown). The effect of time, *b* = -83 (95% confidence interval, -104 to -64), *t* (126) = -8, *p*<0.001, was highly significant, indicating that the DHA status significantly changed over the 15 month study period. Adding a random slope to the model, allowing for interindividual differences in rate of change, did not improve the fit. However, adding time as a quadratic fixed effect improved the fit of the model significantly, *b* = 17 (13, 20), *t* (126) = 9, *p*<0.001. Thus, a random intercept and a second order polynomial best described the trend in the data. The model reflects an initial decrease in the DHA status starting in gestational week 28 with a subsequent increase after about 6 months postpartum (Fig 2B). The second order polynomial gave the function *y* = *x* (-84 (time)) + (17 (time^2^)), where x is the initial DHA status (μg/g RBC) in pregnancy.

Likewise, there were significant between-subject variations in the average LA status (μg/g RBC) over time points (data not shown). The effect of time, *b* = -140 (-2, -94), *t* (124) = -6, *p*<0.001, was highly significant. Adding time as a quadratic fixed effect improved the fit of the model significantly, *b* = 19 (1, 28), *t* (124) = 5, *p*<0.001. Thus, a random intercept and a second order polynomial, or a quadratic trend best described the data (Fig 2C). The second order polynomial gave the function *y* = *x* (-140 (time)) + (19 (time^2^)), where x is the initial LA status (μg/g RBC) in pregnancy.

There were significant between-subject variations in the average AA status (μg/g RBC) over time points (data not shown). The effect of time, *b* = 269 (124, 414), *t* (123) = 4, *p*<0.001, was highly significant, indicating that the AA status significantly changed over the 15 month period. Adding time as a quadratic-, *b* = -126 (-192, -59), *t* (123) = -4, *p*<0.001, and a cubic fixed effect, *b* = 17 (8, 26), *t* (123) = 4, *p*<0.001, significantly improved the fit of the model. Thus, the trend in the data indicates an initial increase with a subsequent decrease and a following second increase in the AA status (μg/g RBC) from pregnancy towards 12 months postpartum (Fig 2D). The third order polynomial gave the function *y* = *x* (269 (time)) + (-126 (time^2^)) + (17 (time^3^)), where x is the initial AA status (μg/g RBC) in pregnancy.

### Seafood consumption

Multilevel linear models were also used to study any change in the seafood consumption over the 15-month time period, from pregnancy until 12 months postpartum. The median weekly frequency of seafood as dinner was 1.5 (0.2–3.0) in pregnancy, and 1.5 (0–3.0), 1.5 (0.2–3.0) and 1.5 (0.2–3.0) 3-, 6- and 12 months postpartum.

There was no significant between-subject variation in the *seafood as dinner* consumption over the 15-month period. However, *age* predicted seafood dinner consumption positively (*b* = 0.03 (0.01–0.06), *t* (102) = 2.6, *p*<0.05) and *parity* predicted seafood dinner consumption negatively (*b* = -0.13 (-0.25–0.005), *t* (102) = -2.1, *p*<0.05). This indicates that for each ten years of ageing between 19 and 42, this study population increased their weekly seafood dinner consumption with 0.3 (0.1–0.6) times. The median weekly frequency of *seafood as spread* was 0.5 (0.2–3) in pregnancy, and 0.5 (0–3.0), 0.5 (0–3.0) and 1.5 (0–3.0) 3-, 6- and 12 months postpartum. There were significant between-subject variations in the seafood as spread consumption over time. Adding time as a quadratic fixed effect improved the fit of the model significantly, *b* = 0.1 (0.04, 0.2), *t* (204) = 3, *p*<0.01. *Age*, *parity* or *education level* did not predict the seafood as spread consumption. The median weekly frequency of *EPA/DHA-supplement* intake was 7 (0–7) in pregnancy, and 5 (0–7), and 2.5 (0–6.5) 3- and 12 months postpartum. There were significant between-subject variations in the *EPA/DHA-supplement* intake Adding time as a cubic fixed effect improved the fit of the model significantly, *b* = 0.8 (0.3, 1.3), *t* (142) = 3, *p*<0.01. *Age*, *parity* or *education level* did not predict the EPA/DHA-supplement intake.

### The impact of breastfeeding on the DHA status

“The DHA status (μg/g RBC) did not differ between women that were breastfeeding (T2: 133 (65, 215) μg/g RBC, T3: 145 (52, 263) μg/g RBC, T4: 163 (56, 208) μg/g RBC) and women that were not breastfeeding (T2: 124 (54, 180) μg/g RBC, T3: 136 (43, 187) μg/g RBC, T4: 170 (116, 220) μg/g RBC), *U* = 453, *z* = -1.3, *ns*, *U* = 670, *z* = -0.9, *ns*, *U* = 723, *z* = 1.4, *ns*, respectively”.

### Predictors for DHA, LA and AA status

The *total seafood-index* significantly predicted the DHA model in a positive manner (*b* = 4 (3–6), *t* (124) = 5, *p*<0.01). Based on our model an increase in total seafood-index by one unit yields an increase in DHA (μg/g RBC) by 4 (3–6) μg/g. Confounding variables including *breastfeeding*, *age*, *education level* and *parity* did not affect the DHA status. There were significant between-subject variations in the total seafood index over the 15-month period (data not shown). *Age*, *parity* or *education level* did not predict the total seafood index.

The AA status was not predicted by the *total seafood-index*, *age*, *parity* or *education level*. However, the *education level* negatively predicted the LA status (μg/g RBC) *b* = -11 (-22, -1), *t* (96) = -2, *p*<0.05), indicating that for each level of education ranging from lower secondary school to four years or more with higher education, the LA status decrease by 11 (1–22) μg/g RBC.

## Discussion

The results from the present study provides novel data regarding maternal DHA stores which decrease after birth and are determined by the DHA status in pregnancy. The maternal seafood consumption and intake of EPA/DHA-supplements positively predicted the DHA status in pregnancy and postpartum. To our knowledge, this is the first study that presents the time course of the maternal FA status during pregnancy and the first year postpartum associated to seafood consumption. An important feature of this study was that all detected FA were submitted to analyses and DHA, AA and LA were among the FA whose abundance profiles varied the most over the 15-month period. For all three the amount in RBC decreased from pregnancy towards six months postpartum, however, different factors predicted the observed changes in FA statuses, respectively.

The maternal DHA status decreased from pregnancy towards three months- and reached a minimum six months after birth. As suggested by Houwelingen *et al*., the DHA status was normalized within one year after the last partus [28]. The decline in maternal DHA status could result from fetus accretion of DHA *in utero*, and lactation during the first six months postpartum. Three months postpartum 81% were breastfeeding daily. Six months postpartum, this was reduced, however only by 11% to 70%. However, there was no difference in the DHA status between lactating and non-lactating women at any measured time point. Neither did breastfeeding effect the overall change in the DHA status over time. Another possible contribution to the decreased DHA could be a change in the dietary habits, decreased consumption of fish in particular. The *seafood as dinner* intake was unchanged during the 15-month period. However, the *seafood as spread* intake, which mainly consists of oily fish, decreased from pregnancy to three months postpartum. The consumption of *seafood as spread* was significantly correlated with RBC DHA in pregnancy (published elsewhere) [24]. Thus, this change in dietary habit could have contributed to a decrease in RBC DHA. Conversely, the *EPA/DHA-supplement* intake, which also correlates with RBC DHA [24], increased from pregnancy to three months which could outweigh net dietary intake of EPA and DHA. The decrease in DHA concentrations is not necessarily explained by fetal and infant accretion and maternal dietary intake alone [29]. Women have a better ability than men do to synthesize DHA from its precursor and it is proposed that these differences could be due to the estrogenic effect to up-regulate delta-6 desaturase [30, 31]. Absolute and relative amounts of plasma and RBC DHA, AA and LA have a net increase during pregnancy [3, 32]. A limitation in this study is that we did not sample blood at birth or the first weeks postpartum. If this had been done, we might have noticed that the drop in RBC abundance of DHA, AA and LA first happened after birth, simultaneously with the major drop in estrogen. If circulating estrogen which rises during pregnancy aid conversion of ALA to DHA during gestation [33] this would end after birth due to hormonal changes. Large variation among pregnant women in plasma DHA concentration at term indicate a metabolic basis in addition to any dietary effects [34]. Since prolactin suppresses estrogen activity, the activity of the desaturation/elongation pathway may be down regulated in lactating women compared with non pregnant and pregnant women [33]. Therefore, the fluctuations in the maternal DHA, AA and LA status during pregnancy and the first year after birth may be due to normal physiological changes. In addition, individual differences in the FADS genotype might have an impact on the variation of DHA levels between mothers.

Nevertheless, although women have physiological mechanisms to adapt to the high demands of DHA [3, 35] during pregnancy, results from the present study showed that the higher the intake of seafood and EPA/DHA-supplements were, the higher the DHA status in the maternal circulation. As this is a small longitudinal observational study, it was not possible to assess differences between women with a high seafood intake and women with a low seafood intake. However, results from a dietary intervention study performed by Miles *et al*. [36] indicated that women consuming two portions of oily fish per week, during pregnancy, did not suffer from pregnancy-associated decline in DHA. In fact, the plasma DHA status in the intervention group increased throughout pregnancy. Concurrently, in an intervention trial, women who received 200 mg DHA from mid pregnancy through lactation had significantly higher RBC-DHA% than the control group [37]. This underlines the importance of consuming sufficient amounts of DHA during pregnancy, as an increased synthesis of DHA might not be adequate to support the high demands of the fetus and at the same time ensuring sufficient maternal requirements. The total intake of EPA and DHA from seafood consumption and supplements, as monitored in this study, did not increase significantly from pregnancy to twelve months postpartum. However, EPA/DHA-supplement intake decreased and *seafood as spread* consumption increased. It might be presumed that women increase their seafood intake after pregnancy and lactation as the fear of transferring undesirable substances that might be present in seafood to the developing fetus and infant is reduced [38].

In order to determine whether an individual has a low DHA status, absolute amounts of RBC DHA (μg/g) in non pregnant, non lactating premenopausal women could be used as a reference. We used the two and a half percentile DHA status twelve months after delivery as a proxy, detecting the percentage of women with low DHA status in pregnancy, and three and six months after birth. The lowest values were measured three and six months after birth when about one third of the participants had RBC DHA status below the two and a half percentile, which corresponds to an n-3 index of just below 4% in pregnancy [8]. These results coincide with the quadratic trend for overall change in DHA status modeled from this study. The n-3 index is a marker of increased risk for death from coronary heart disease, [7, 39]. Optimal levels appear to be 8% or greater. [12]. In a previous publication from the present study, we correlated the omega-3 index with the absolute amount of DHA in the same samples [8]. An omega-3 index in pregnancy of 5% or below was associated with an increased risk of postpartum depression six months later. Thus, we have reason to suggest that the low DHA status’ detected at three and six months after birth in this study may be below adequate levels for the mother. Nevertheless, the relative amount of RBC DHA found in this study is three times higher than the one found in a large cohort from the UK [12] and about 30% higher than the one found in a group of Dutch pregnant women [40], perhaps because of a higher habitual seafood consumption in Norway.

In the present study, the LA status, which decreased from pregnancy to postpartum, was negatively associated with education. Conversely, AA, which did not decrease before after 3 months postpartum, was not affected by any of the predictors. LA is predominantly found in processed foods with a high content of vegetable oils, soybean oil in particular. Excessive dietary intakes of LA may promote obesity [41]. In the U.S., the apparent increased consumption of LA has likely decreased tissue concentrations of EPA and DHA during the 20th century [42]. In the Norwegian Mother and Child Cohort Study [43], education was also negatively associated with a dietary pattern high in processed foods [44]. Interestingly, this dietary pattern was also negatively associated with consumption of both oily- and lean fish. Consequently, less educated women may have a n-3/n-6 ratio that is lower due to both lower dietary intake of n-3 FA and higher dietary intake of n-6 FA. Even though an optimal AA-status is important for human health, the intake of n-6 FA’s is more than sufficient in developed countries [45]. In fact, a lower consumption of LA could reduce the enzymatic competition and lead to an increased conversion of ALA to EPA and DHA.

Despite large variation in the DHA status the decrease in RBC DHA between pregnancy and three months post-partum was found to be significant. The observed decrease is likely due to normal biological changes causing an altered DHA metabolism in pregnancy and breastmilk production/lactation. Without clinical reference values we do not know which potential consequences a lower DHA status can lead to. Using the DHA status one year after birth as a reference, it was found that approximately one third of the women displayed DHA levels below the 2.5 percentile three and six months after birth, respectively. Observational studies and clinical trials have evaluated the possible role of omega-3 fatty acids consumption in the etiology of postpartum depression and suggest an association between low levels of marine omega-3 fatty acids and the occurrence of depression in the postpartum period [46–51]. Moreover, epidemiological studies show that higher intakes of EPA and DHA from seafood during pregnancy improved developmental outcomes in the offspring [10, 52]. Further studies are needed to address weather a decreased DHA status postpartum, and at which level, poses negative consequences for the mother or the child.

## Limitations

The present study faced challenges due to recruitment issues at both T1 and T2 (28^th^ gestational week) and T2 (three months postpartum) resulting in an untraditional longitudinal study design. The rationale for further recruiting after T1, the cohorts first and most important time point, was to represent a wide spectrum of women form the source population to minimize selection bias and maintain statistical power of the analysis, respectively. However, while inevitable, the additional recruiting may weaken the conclusions to be drawn from the longitudinal analysis performed in the present study. By chance the DHA status of participants entering the study at T2 could have had been very different to the DHA status of those participants who entered at T1. If this were the case, the significant decrease in DHA status from T1 to T2 would therefore be in parts due to new participants’ entering the study. To address this limitation, we compared participants whom we sampled blood from at all four time points and those we did not at each time point. There were no significant differences in the relative or absolute amount of LA-, AA-, or DHA in the RBC between the two “groups”. Thus, there is reason to believe that participants recruited at T2 do not differ to those recruited at T1 in their pregnancy DHA status. Thus, while not ideal, we believe that the additional recruiting did not invalidate the study design or the conclusions to be drawn.

## Conclusion

Our results suggest that the maternal DHA status in pregnancy is pivotal for the DHA status the first year postpartum. The continuous decrease of maternal DHA status from pregnancy until six months postpartum may suggest that the maternal capacity to meet the high fetal requirements for DHA is inadequate. The functional implications of pregnancy-associated reduction in the maternal LA, AA and DHA status for fetal and neonatal development need further studies. In addition, the results indicate that increasing maternal seafood consumption or EPA/DHA-supplement intake during pregnancy may be beneficial to the mother and child ensuring sufficiently high DHA levels. These findings further indicate that promoting adequate maternal dietary intake of DHA before, during and after pregnancy is important to maintain sufficiently high levels of DHA that meet the requirements of fetal and infant development as well as requirements of the mother.
